# Survey on the knowledge, perceptions, and concerns of parents about childhood vaccinations in Italy

**DOI:** 10.3389/fpubh.2026.1950327

**Published:** 2026-10-27

**Authors:** Laura Teodori, Eugenio Serravalle, Franco Stocco, Panagis Polykretis

**Affiliations:** 1 AsSIS Associazione di Studi e Informazione sulla Salute - The Association for Studies and Information on Health, Pisa, Italy; 2 Independent Medical-Scientific Commission, Milan, Italy; 3 “Allineare Sanità e Salute” Foundation, Milan, Italy; 4 Ancestralize Ltd., Üröm, Hungary

**Keywords:** childhood vaccination, national opinion, professional information, scientific information, survey

## Abstract

**Objective:**

The present study aims to assess Italian families' knowledge, perceptions, and concerns about children's vaccinations through a national survey among 1,000 families with children aged 2 months to 16 years, stratified by sex, age, and area of residence. The overall margin of error was ±3.1%, and responses were also examined by geographical area, self-assessed socioeconomic status, and parental age.

**Results:**

Most parents (90%) consider pediatric vaccines essential for their child's health, 86.5% recognize their importance for public health, 73% parents trust vaccines safety. However, only 34% correctly identified mandatory vaccines and just 17% distinguished between mandatory and recommended ones. Responses regarding compliance with vaccination requirements for school enrollment show an even higher level of disinformation, or incorrect/uncertain responses.

**Conclusions:**

Despite the high level of knowledge about vaccines declared by the respondents, their answers do not indicate a very deep knowledge. The findings of the survey highlight concerns on the quality of information and the limitations of information provided by Italian institutional sources and national media showing responses that varied systematically by political orientation, indicating knowledge gaps rather than being uniformly grounded in scientific and legislative information. The necessity for more in-depth and comprehensive information is indisputable and vaccine information for parents should have vaccine safety and efficacy at its core.

## Introduction

The U.S. Centers for Disease Control and Prevention recommends a Child and Adolescent Immunization Schedule by Age (1). In compliance with the advice of the CDC, in Italy, the Legislative Decree of 7 June 2017, n. 73, amended by the Conversion Law of 31 July 2017, n. 119 (2), (called Legge Lorenzin, from the Health Minister who presented the law) provides for 10 compulsory vaccinations and 4 recommended vaccinations for minors aged between 0 and 16 years (3). Already in 2014, as part of the Global Health Security Agenda, Italy was designated as the world outpost in vaccination strategies and campaigns.

The Italian Ministry of Health advises that all children follow the CDC recommended immunization schedule. The Italian vaccination schedule is included in the National Vaccine Prevention Plan (PNPV 2023–2025) and approved by the State-Regions Conference (4). Thus, since 2017, 10 vaccines are required by law for children under 16 years of age following a Vaccination Calendar schedule. The mandatory vaccines are: anti-polio; anti-diphtheria; anti-tetanus; anti-hepatitis B; anti-whooping cough; anti-haemophilus influenzae type B; anti-measles; anti-rubella; anti-mumps; anti-chickenpox. Four other vaccines (i.e., pneumococcal conjugate vaccine, PCV; rotavirus; B and C meningococcal vaccines) are strongly recommended and free of cost (5).

Pediatric vaccine formulations are usually combination vaccines containing multiple antigens provided with a single injection. Examples of these formulations include the hexavalent vaccine, against diphtheria, tetanus, pertussis, polio, hepatitis B, and Haemophilus influenzae type b; the trivalent MMR vaccine, for measles, mumps, and rubella; and the tetravalent MMRV vaccine, for measles, mumps, rubella, and varicella. Other formulations include the pneumococcal conjugate vaccine (PCV) and the meningococcal B vaccine (MenB). The calendar vaccination schedule is reported on the Ministry of Health's web portal (6).

Compliance with the vaccination requirements is a condition for admission to kindergartens and nursery schools (for children aged 0 to 6 years), while from primary school onward children and adolescents can continue to attend school and take exams, but if the requirements are not met, a vaccination recovery process is activated by the local health authority and administrative fines of between 100 and 500 euros can be imposed.

The situation in Europe is somewhat different. Each EU/EEA country is responsible for its own national public health policy, including its national immunization program and schedule (7). Each country's immunization program may differ. The differences concern the age of the population to be vaccinated, including population groups, the number and timing of doses, the decision to give one vaccine alone or in combination with others, the extent of obligation. Indeed, there are 17 European countries that have no mandatory vaccinations, while 13 countries have one or more mandatory vaccinations in their vaccination program (8). Notably, this landscape is not static: between 2014 and 2024, six countries (Croatia, France, Germany, Hungary, Italy, and Poland) introduced or extended mandatory childhood vaccinations, reflecting a broader European trend toward increased vaccination obligations (8).

With the aim of assessing the knowledge, judgments, perceptions, fears, and concerns of Italian families with children aged 2 months to 16 years, we commissioned a survey on the level of knowledge of which vaccinations are mandatory and which are recommended in Italy; which vaccinations are necessary to attend nursery school, primary school and secondary school; what the constitutional obligations are; the safety of vaccines and the risks of adverse events and we compared answers with their political orientation. Comparisons with obligations in Europe were also explored.

## Methods

This survey was performed by Euromedia Research, one of Italy's most important market research institutes able to manage any research project, from conception to methodology, from information gathering to statistical analysis and data interpretation, to presentation and definition of marketing strategies (9). The survey was commissioned by the AsSIS (Health Studies and Information Association) (10), a scientific association made up of physicians, scientists, health professionals and lawyers who work to promote the disease prevention and care of human beings, maintaining a position of total autonomy toward any organization, school, movement, trend or interest group that could potentially affect health policy decisions or conduct medical research.

### Study design

The survey was conducted using C.A.W.I. (Computer-Assisted Web Interviewing) methodology. The advantages of this questionnaire-based methodology, which involves providing respondents with a link, a panel or a website, are illustrated in CAWI Methodology (11). The sample of 1,000 interviews was stratified by area of residence, gender and age, with an overall statistical error of ±3.1%. This margin of error is consistent with the standard formula for a proportion estimate at the 95% confidence level and a sample size of 1,000. This margin of error applies to estimates derived from the overall sample and does not extend with the same precision to subgroup analyses, particularly smaller political-affiliation categories, for which sample sizes and corresponding precision are reported where relevant (see also Limitations). The reference data used to construct the sample were based on households with children, with the youngest child being under 17 years of age, adjusted for sex and age (processed data from ISTAT 2023). The sample was defined as illustrated in Figure 1.

**Figure 1:**
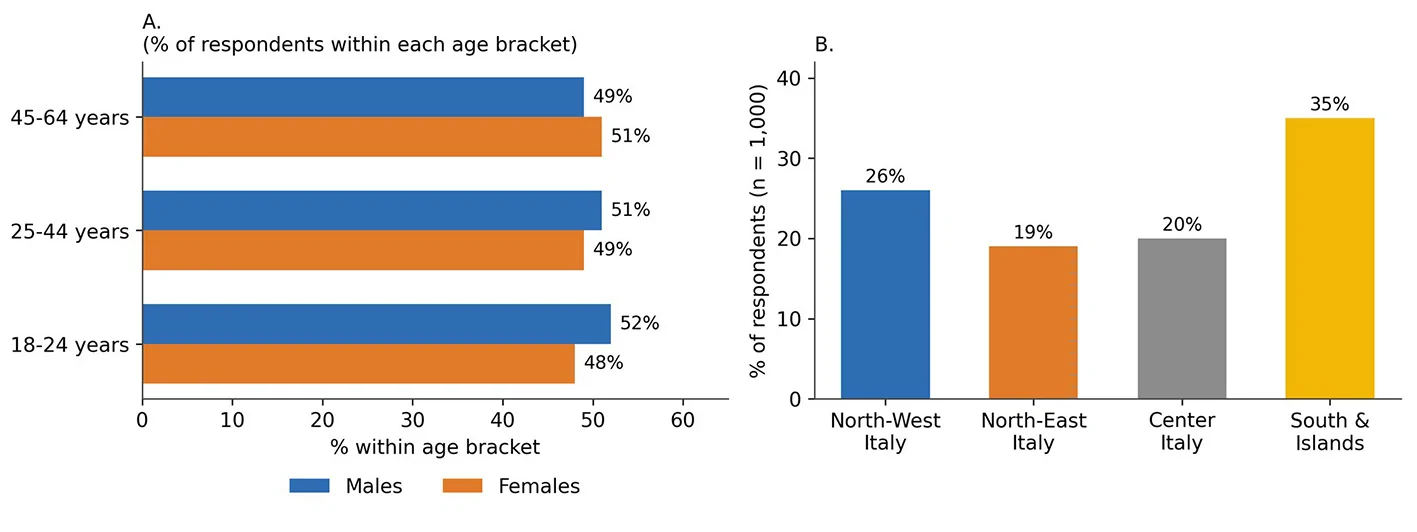
Definition of the interviewed sample based on years and gender **(A)**, and the geographical area of the respondents **(B)**.

Quota targets for each stratum (geographical area, gender, age) were set a priori based on ISTAT 2023 household data and monitored by the agency during fieldwork; interviews were closed once quotas were filled, a standard procedure intended to approximate population representativeness without *post-hoc* weighting.

The questionnaire was developed *ad hoc* for the purposes of this study by the commissioned research agency (Euromedia Research), in consultation with the authors, and was not adapted from pre-existing validated instruments. Knowledge-based items were constructed to reflect current Italian vaccination legislation (Legislative Decree 73/2017 and PNPV 2023–2025), and correct answers were determined by the authors on this basis. Items were formulated in clear, accessible Italian and underwent a preliminary comprehensibility check prior to administration; a formal cognitive pilot study with a separate sample was not conducted. No formal assessment of reliability or validity was performed. The complete questionnaire is provided in the Supplementary material.

All data were processed and analyzed using IBM SPSS Statistics. Descriptive statistics (absolute frequencies, percentages, and means, as appropriate) were computed for the overall sample and for each subgroup (parental gender and age, children's age, geographical area, and political affiliation). No inferential statistical tests, multivariable regression models, or corrections for multiple comparisons were performed; reported percentage differences between subgroups are descriptive rather than the result of formal statistical hypothesis testing. Political affiliation was assessed via a single self-report item asking respondents to indicate their current voting intention among a list of Italian political parties/coalitions (see Supplementary material for full item wording); respondents were categorized accordingly, with smaller parties aggregated into a “minor parties” category for reporting purposes.

### Survey execution

The sample consisted of parents, of both sexes, aged between 18 and 64, with at least one child aged 0 to 16 (the age range covered by the main childhood vaccination schedules). This ensured that respondents were directly involved in decisions regarding their family's health.

A total of 2,982 contacts were made. Of these, 1,000 interviews were fully completed, while 1,982 contacts resulted in refusals, corresponding to 66.5% of total contacts. Incomplete interviews and refusals were excluded from the final sample. Given the completion rate of 33.5%, the possibility of non-response bias cannot be excluded; this is addressed in the Limitations section. As the resulting sample matched target quotas on area, gender, and age, no additional weighting adjustments were applied; however, representativeness on other unmeasured variables (e.g., education, socioeconomic status, health literacy) could not be verified.

The final dataset of 1,000 interviews includes only respondents who answered all questions, ensuring full compliance with the survey quotas. Item-level “*Don't know/No answer*” responses were retained as a distinct response category in the reported results, rather than treated as missing data. Responses were analyzed and grouped according to parent's age, parent's gender, child's age, geographical area of residence, and political affiliation.

### Survey administration process

1. An email was sent, including an introduction letter about the research (the research sponsor remained anonymous) and an active link to access the questionnaire.
2. By clicking the link in the email, respondents were directed to a web platform, where they could complete the survey online.
3. Upon completing the questionnaire, a thank-you message appeared on the screen.

It must be noted that only 33.5% of interviewed parents agreed to respond.

## Results

The data of the survey clearly shows the extent to which parents consider vaccination to be essential for the health of their child (90.1% of the sample vs. 9.8% who consider it to be of little or no importance) and for the health of the community (86.5% vs. 12.6%). The overall responses are illustrated in Figure 2 for the child's health and for the community, respectively.

**Figure 2:**
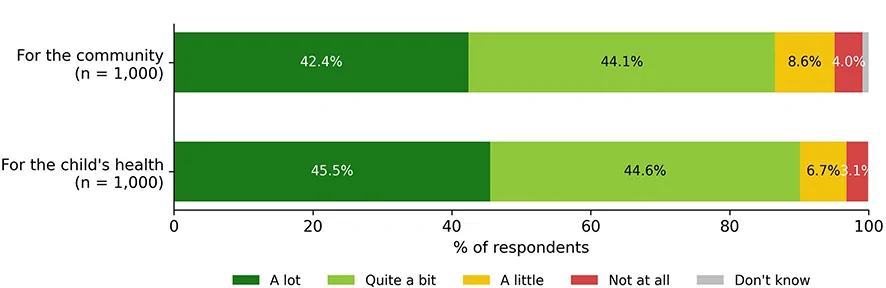
Perceived importance of pediatric vaccinations for the child's health and for the community, overall sample (*n* = 1,000). Exact percentages are labeled for each response category.

There is not much difference in the answers according to the sex and age of the parents, or the age of their child. The detailed information is reported in Supplementary Table 1 for child health, and Supplementary Table 2 for community health, respectively, according to age, gender and area of residence.

This survey also evidenced large differences of opinion in terms of political orientation. A large majority of Democratic Party (PD) voters (97.2%) and of Greens and Left Alliance (AVS) voters (96.3%) are more in favor of the benefits of vaccinations for the child and the community, as respect for example to the 85.4% of minor parties for child health and 80.5% for community health (see Supplementary Tables 1.1, 2.1). For comprehensive information on Italian parties, see the list of political parties in Italy (12). A graphic representation of the answers is illustrated in Figures 3A, B for child and community, respectively. The detailed information is illustrated in Supplementary Tables 1.1, 2.1.

**Figure 3:**
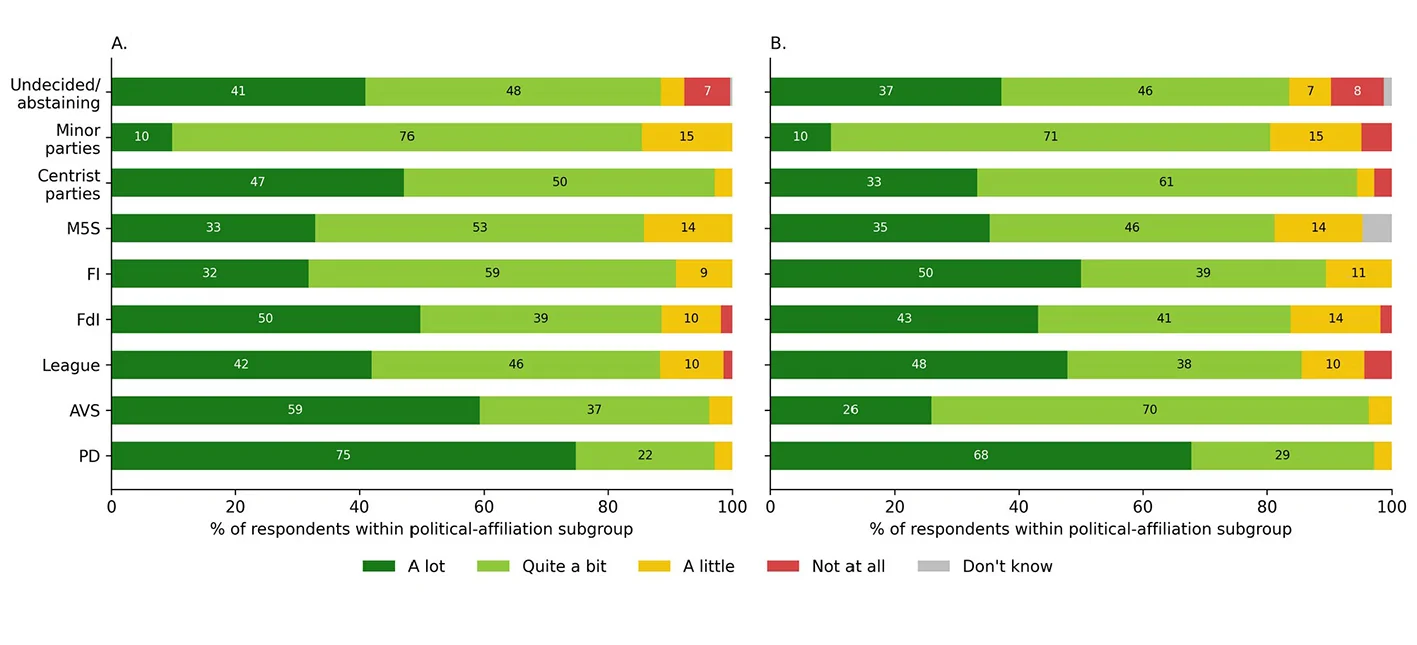
Perceived importance of pediatric vaccinations for the child's health **(A)** and for the community **(B)**, by parental political affiliation. Values are row percentages within each political-affiliation subgroup, as reported by the survey agency; subgroup sample sizes (*n*) were not provided by the agency (see Limitations).

When asked whether they were in favor of or opposed to mandatory vaccination against certain diseases for minors under 16, 74.0% of the parents surveyed were in favor, especially of the vaccinations currently required by law (49.3%). Noteworthy, around one in four parents (24.7%) also want childhood vaccinations that are currently recommended to be made mandatory. The responses are graphically illustrated in Figure 4A and the raw data are illustrated in Supplementary Table 3.

**Figure 4:**
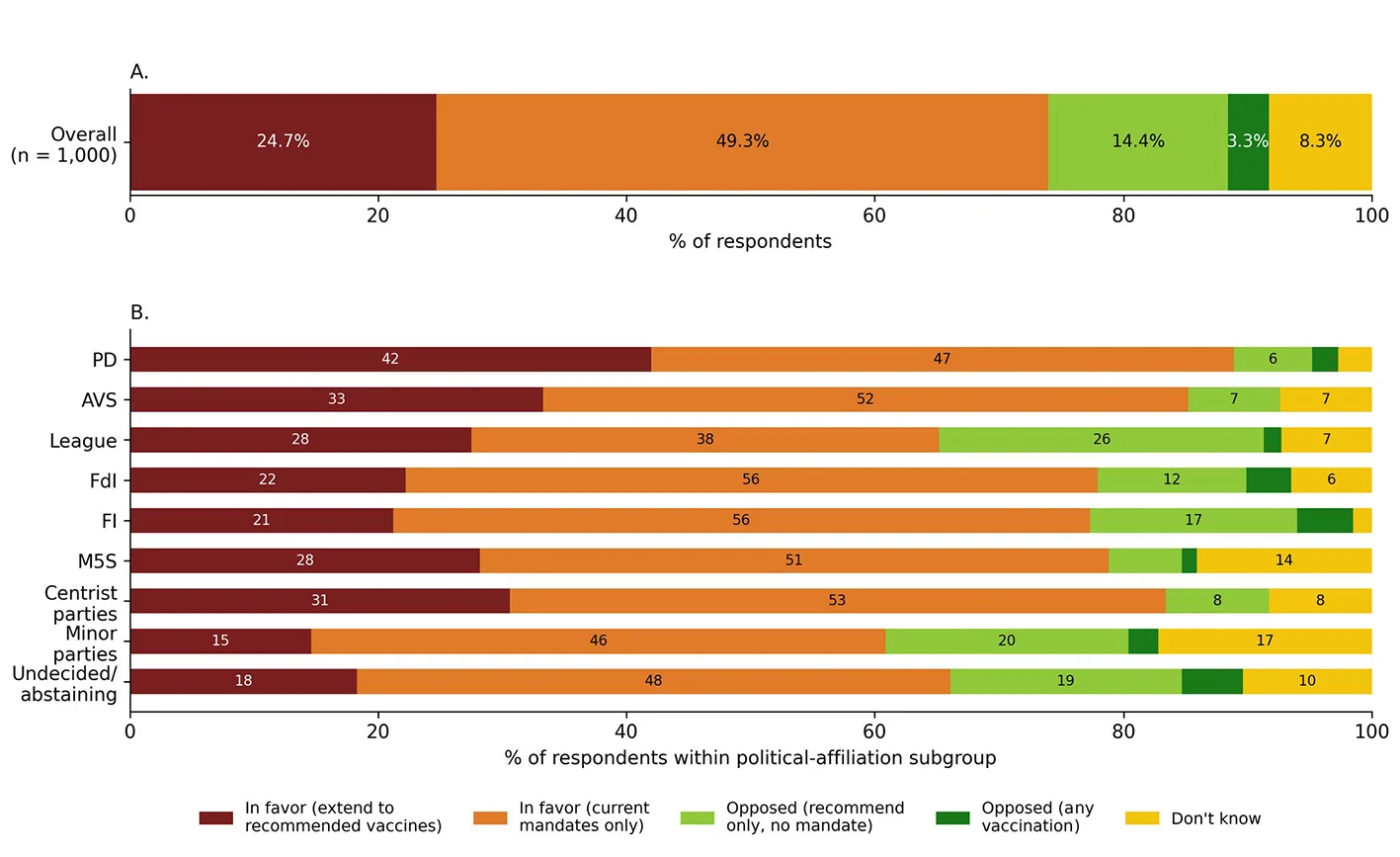
Support for mandatory childhood vaccination, overall (**A**, *n* = 1,000) and by parental political affiliation **(B)**. Panel **B** values are row percentages within each subgroup; subgroup sample sizes (*n*) were not provided by the survey agency (see Limitations).

As respect of parents age and gender, those most in favor are men, aged between 45 and 64, with children under the age of 2, living in southern Italy and in the islands.

Regarding political affiliation, PD voters (88.9%) and AVS voters (85.2%) stand out among those in favor of mandatory childhood vaccinations; the most skeptical are the supporters of the small parties (60.9%), the League (65.2%) and the undecided (66.1%), as illustrated in Figure 4B and Supplementary Table 3.1.

The above data show a high prevalence of positive vaccination approach responses, however a lack of knowledge about several important aspects, including mandatory or recommended pediatric vaccines is observed in following questions. Indeed, there is a lot of misinformation, or limited knowledge about which childhood vaccinations are mandatory (M), and which are recommended (R) in Italy. Regarding the (M), results are reported in Supplementary Table 4 (age, gender, children's age) and Supplementary Table 4.1 (political orientation). Regarding the (R), results are reported in Supplementary Table 5 (age, gender, children's age) and Supplementary Table 5.1 (political orientation). Less than 50% of respondents know the correct answer, while 13.4% (M) and 18.5% (R) do not know the answer at all; a significant proportion of respondents even consider the recommended vaccinations to be mandatory. Conversely, there are mandatory vaccinations, such as against measles and rubella, which are incorrectly recognized as recommended by 21.0% and 20.3% of respondents, respectively. About the recommended vaccinations, the highest correct answer was given by 22% only of respondents. A synthetic overview of these results is provided in Supplementary Tables 6, 6.1.

Once again, the data show that there are greater differences in political affiliation than in age, gender of parents, and age of their children. These misconceptions, or knowledge gaps, become even more evident when the issue of vaccination compliance is introduced as a requirement for access to different levels of schooling (Supplementary Tables 7–11). Only 35.2% of respondents correctly state that vaccination is required for enrollment in nursery schools and pre-school (Supplementary Table 7); in fact, in Italy, enrollment in nursery schools and pre-school (for children aged 0–6) is conditional on compliance with vaccination requirements. Children over 6 and adolescents can continue to attend school and take exams, but if the requirements are not met, a vaccination recovery process will be activated by the local Health Authority and administrative fines between 100 and 500 Euros may be imposed. However, as much as 27.8% believe that vaccination is essential for enrollment in any level of school. Finally, 5.9% of parents believe that it is not necessary to vaccinate their children to enroll them in any school.

The percentages relating to “Does not know/Does not answer” increases with increased school level up to 39.0% for secondary school (children aged 11–16 years). The answer to “No vaccination is mandatory” also grows from one school level to the next: for nursery and nursery school it stands at 7.6%, for primary school at 14.6%, for lower secondary school at 19.5% and for upper secondary school at 22.7%. A summary of these data across school levels is provided in Supplementary Table 12. It is worth noting the difference in responses when political orientation is considered.

Knowledge of childhood vaccination requirements in the rest of Europe is also very low. Indeed, when asked about the situation of mandatory childhood vaccinations in the rest of Europe, only 16.1% of the sample correctly said that many European countries have fewer mandatory vaccinations than Italy, or none at all, while as many as 38.6% said that most of other European countries have the same rules as Italy and 21.5% said that about half of the other European countries have mandatory childhood vaccinations as Italy. Results are illustrated in Supplementary Table 13.

An overview of obligations and recommendations in Europe and vaccination coverage is given in Vaccination schedules in the EU/EEA (7). In summary, 17 countries have no obligations at all (Austria, Greece, the Netherlands, Sweden, Norway, Liechtenstein, Ireland, Finland, Lithuania, Portugal, Estonia, Romania, Iceland, Denmark, Luxembourg, Spain, and Cyprus). Germany for measles only and Belgium for polio only. Malta only for diphtheria, tetanus, pertussis, and polio. Latvia holds the record with 13 mandatory vaccines.

When it comes to trust in vaccines, nearly 3 out of 4 (73.1%) respondents are in favor of childhood vaccines as a safe health tool. A graphical representation is illustrated in Figure 5.

**Figure 5:**
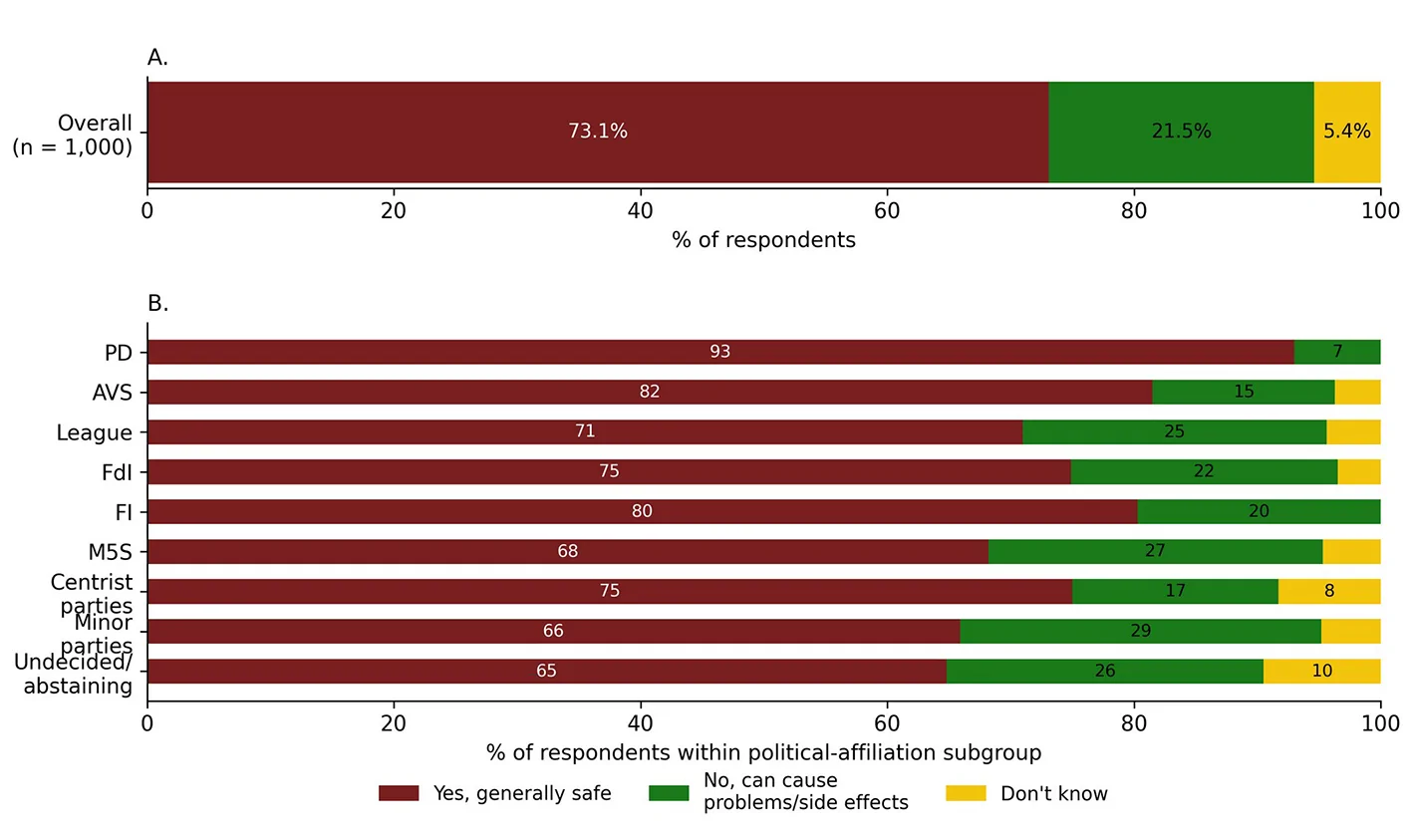
Perceived safety of pediatric vaccines, overall (**A**, *n* = 1,000) and by parental political affiliation **(B)**. Panel **B** values are row percentages within each subgroup; subgroup sample sizes (*n*) were not provided by the survey agency (see Limitations).

As illustrated in the figure, 93.0% of PD and 81.5% of AVS voters responded that childhood vaccines are a safe health tool vs. 71.0% of League for Salvini and 68.2% of M5S. Noteworthy is the low percentage to question “Yes, they are generally safe” in politically undecided or abstainers the raw data are reported in Supplementary Table 14.

In Supplementary Table 14 the answers are reported in terms of demographic variables. The feeling of greater “insecurity” is perceived by mothers (23.8%), those with children aged 2 to 6 (25.3%) and those living in the north-east (27.1%). The perception of the safety of pediatric vaccines is very high among those with children aged up to 2 years (83.2%), i.e., those who have recently come into contact with the world of pediatric vaccination.

Respondents were then asked to agree or disagree with a statement about the danger or otherwise of vaccines: 70.7% of the sample disagree that “childhood vaccines are more dangerous than the diseases they target”, while just over 1 in 4 parents (27.4%) believe the opposite. The data are reported in Supplementary Table 15. Once again, there are polarized responses when it comes to political orientation. In Supplementary Table 15.1 is clear that the PD voters who agree with the question “Pediatric vaccines are more dangerous than the diseases to which they are directed” are less than a quarter of League voters, the answers being 11.9% and 46.4%, respectively.

For contextual purposes, we compared overall parental attitudes toward vaccination with the 2023 (last updating) national vaccination coverage (VC) (13), to provide broader context on actual vaccine uptake in Italy alongside the survey's attitudinal findings, without implying that population-level coverage validates individual-level response accuracy. Polio (IPV) coverage, used as a proxy for hexavalent vaccination (diphtheria, tetanus, whooping cough, hepatitis B, polio, and disease caused by Haemophilus influenzae type b) showed a coverage of 94.76%. MPR (measles-mumps-rubella) first dose 94.64%, second dose 84%−86%. It is worth noting that the second dose of the MPR vaccine has been mandatory in Italy since 2017, with the second dose administered at 5–6 years of age. This explains the lower coverage (84%−86%) compared to the first dose, as not all children will have reached this age by 2023.

## Discussion

The results of this study are highly relevant in the context of the ongoing political and public debate about children mandatory vaccination. This survey provides important insight into parents' vaccine knowledge and highlights the areas that remain unclear. Most published survey studies have as primary focus vaccine hesitancy (VH), as confirmed by the high number of studies and questionnaires found in the literature on the matter (14). A systematic review draws attention to the fact that the concept of VH has been described and applied in somewhat inconsistent ways (15). E.g., parents seeking more information to make “the right decision” about vaccination are often defined as hesitant or because perceiving ambiguity in vaccine-related risks. Some other studies also focus on the development of practical tools to better estimate vaccine hesitancy (16). Other surveys attempt to monitor the decline in vaccine confidence compared to previous years (17). Following an in-depth review of the VH literature, we argue that vaccine hesitancy cannot be reduced to misinformation and diminished disease risk perception alone, as some authors have proposed (8).

It is important to distinguish between related but non-interchangeable constructs used in this literature. Vaccine hesitancy refers to a state of ambivalence (delaying or accepting vaccination with reservations); vaccine confidence refers to trust in the safety, efficacy, and reliability of the health system delivering vaccines; vaccine acceptance refers to the behavioral outcome of receiving or intending to receive a vaccine; and factual vaccine knowledge refers to accurate awareness of specific facts, such as which vaccines are legally mandatory.

In this study, we focused on the level of awareness and quality of information that parents receive. Italian Public Services and Health Authorities run information campaigns to encourage parents to vaccinate their children, in accordance with the schedule of the 10 mandatory and 4 recommended vaccines established in the national PNPV (18).

Italy is among the countries with the highest children vaccination rate. A recent report (2023) on 21 countries found that among parents of children under age 6 in Italy, 83.6% reported that their child was up to date on routine vaccines (19), while a companion analysis of the same survey, among parents of children under age 5, found that less than half of respondents in Indonesia (47.6%) reported that their child was fully vaccinated (20).

An analysis of the data from our survey reveals a scenario characterized by a lack of clarity and a certain degree of confusion; a finding that would not be expected among the families of children and adolescents who are the primary stakeholders in pediatric vaccination decisions.

A previous survey conducted in Italy in 2014 on a comparable sample size (1,000 parents), supported by Sanofi Pasteur MSD (21), similarly identified a gap between self-reported and actual knowledge: while 79% of parents declared familiarity with the vaccination schedule, only 5.6% could correctly name the vaccines mandatory in Italy at the time. Such pattern (high declared confidence in vaccine knowledge coupled with limited factual accuracy) closely parallels the findings of the present survey, suggesting that this gap has persisted in the Italian population for at least a decade. The same 2014 survey also reported that institutional and scientific websites (40.6% and 37.2% of parents, respectively) were consulted more often than forums, blogs, or social networks (27.2% and 16.1%, respectively) for vaccine-related information. What our survey adds for the first time is an exploration of parents' political orientation, the consistency and magnitude of which may point to a notable ideological connotation in attitudes toward vaccine culture.

Italy has 10 mandatory vaccinations, one of the highest levels in Europe, alongside France, Hungary, and Latvia, which mandate all but one of the vaccines considered (8). Seventeen European countries do not have any mandatory vaccinations, while Germany and Belgium have only one mandatory vaccination each (8). Thus suggesting, as pointed out from institutional information itself, that the governmental choice is most likely a political choice.

The analysis of the results of the survey commissioned by AsSIS shows that the vaccination obligation is highly supported by the respondents, in fact more than 90% believe that it is beneficial for their children, and these data are in line with the national vaccination coverage updated in 2023. Remarkably, the survey highlighted some critical issues in the three objectives it set itself: Knowledge, Perceptions, and Concerns; these critical issues point to a lack of knowledge that undermines informed and responsible choice among a significant proportion of the population. In the authors' assessment, based on a critical examination of official information—namely, the vaccination calendar and the Ministry of Health's dedicated portal (5)—these sources appear to do little to encourage informed deliberation, and may instead promote blind trust rather than active engagement. As pointed out by Ruth Faden's

“A History and Theory of Informed Consent,” (22)

the deliberative consent should include “*understanding (of all important information), no control (freedom from coercion) and intentionality*.”

Despite the high value of respondents in favor of obligations, misinformation, or incorrect/inconsistent responses, was observed among them regarding several issues. One of these issues concerns the answer to the benefit of vaccines for the community. The health of the community assumes that vaccines are highly immunizing and that the absolute risk-benefit ratio (ARR) is in favor of vaccinations. However, in the authors' view, at least 8 of the current mandatory pediatric vaccines have a more limited effect on population-level (herd) protection than others, without necessarily contributing to interruption of transmission or long-term disease elimination (distinct epidemiological outcomes that should not be conflated with the direct, individual protection). Several of these vaccines either target pathogens not transmitted person-to-person (for which herd immunity is conceptually inapplicable, e.g., tetanus), or induce immunity that wanes over time, or do not fully interrupt transmission even when individual protection is achieved. A list of dedicated references supporting these distinctions, vaccine by vaccine, is reported in references as given in the Supplementary material. In this survey a concept of herd immunity is perceived by respondents as homogeneous between the different vaccines, with the consequent belief that mandatory vaccination is a value for the society for all these products. Indeed, 86.5% of parents believe that vaccination is essential for the health of the community; 24.7% also want the currently recommended childhood vaccinations to be made mandatory. One of the origins of these beliefs is the fact that products with different pharmacological and clinical profiles are grouped together in the same category. Some generic statements in institutional documents also contribute to this general and imprecise perception, such as PNPV 2023–2025 statement:

“All countries recognize vaccination as a priority” or “Vaccines have great value from a human, ethical and social point of view.” (4)

A fragmented perception of the risk-benefit ratio of different vaccines is also observed in this survey, 70.7% of the sample disagree that “childhood vaccines are more dangerous than the diseases they target,” while over 1 in 4 parents (27.4%) believe the opposite. Moreover, 24.7% would also like to see currently recommended childhood vaccinations made mandatory. Contributing to this lack of uniformity of judgment is the absence of information on the absolute risk reduction (ARR) for each product in most scientific publications and communications to the public, where, conversely, the relative risk is provided. This contrasts with the FDA's own guidance on risk communication, which explicitly recommends:

“*Provide absolute risks, not just relative risks. Patients are unduly influenced when risk information is presented using a relative risk approach; this can lead to sub-optimal decisions. Therefore, an absolute risk format should be used*.” (23)

This survey also reveals only partial knowledge of the topics it investigated. For example, in response to the question “From what you know, what are the mandatory pediatric vaccinations in Italy?” (multiple answers were allowed), fewer than 50% of respondents answered correctly. There are mandatory vaccinations such as anti-measles and anti-rubella which are incorrectly recognized as recommended by 21.0% and 20.3%, respectively with a maximum of 29.4%. The inability to access monovalent vaccines nationwide, probably contributes to this inaccuracy; the use of polyvalent formulations (hexavalent and tetravalent or trivalent) leads to an unclear perception of the number of vaccines and doses to be administered for each subject. Even more disinformation, or a larger knowledge gap, is evident when the question regarded recommended vaccination as for example the case of the Anti-meningococcal B and C where more than 25% of respondents believed them mandatory.

Responses based on political orientation revealed even more fragmentation, for example the response to the question “Do you consider childhood vaccines to be a safe health tool” 93% of the Italian Democratic Party (PD) vs. 74.9% of the Fratelli d'Italia (FdI), a national conservative political party, consider pediatric vaccines a safe health tool. This fragmentation of responses along political-affiliation lines, consistent in magnitude and direction across nearly all survey items, may suggest that vaccine-related knowledge and attitudes are influenced, at least in part, by ideological-electoral factors rather than by biomedical and pharmacological information alone; however, given the cross-sectional design and the unavailability of individual-level data for multivariable adjustment, this association cannot be interpreted as establishing a direct causal mechanism, and residual confounding by correlated demographic or socioeconomic factors cannot be excluded. This observation has also been reported in other national surveys whose results suggest that most parents are susceptible to vaccine messages from political and medical leaders (24).

A great deal of unawareness of the provisions of other European States on vaccination obligations is also shown from respondents, more than 60% with a peak 81.1% of PD voters think that obligations are the same in most European countries. Only 16.1% correctly responded that almost all European states have fewer or no obligations at all.

In the authors' view, a detailed look at the information in the PNPV (25), reveals that the recommendations issued by institutions and scientific societies are more reassuring than informative, appearing to emphasize the benefits of a given treatment while giving comparatively less attention to its risks; in the authors' assessment, the citations included in these documents appear to favor research supporting a predetermined thesis, omitting evidence that does not conform to it. Yet, one of the key requirements for mandatory programs to be effective is a reliable supply of safe and effective vaccines (26). Furthermore, because children's vaccines are mandatory and because state law requisite for children is to be vaccinated before being admitted to daycare and school, it is essential that safety concerns be fully and carefully studied.

Despite the widespread assumption that social media is becoming the primary source of health news and information, most parents still consciously rely on official government websites and health professionals for information about vaccines (27). In the authors' assessment, the official channels sometimes underestimated the risk of adverse events. For example, the official channels of the Italian Ministry of Health, and even the FAC portal, lack information based on reliable scientific literature, which raises serious concerns in several respects: there is no mention of the risk of adverse events, the risk-benefit ratio, or side effects (28). In Italy, following the COVID-19 pandemic, a growing popular movement has emerged. Many citizens are demanding greater transparency, based on scientifically approved and clearly shared results. According to this view, only open information can guarantee trust and consensus. At this point, the question arises: is it right to further restrict freedom when citizens are simply asking for transparency and a science based on concrete evidence, and not on ideologies?

A recent paper published in Vaccine, examining public response to the Trump administration's “Make America Healthy Again” (MAHA) vaccine policy proposals, reported that most parents said their trust in vaccine safety would increase if additional vaccine trials were conducted. Even among groups already confident in vaccine safety, additional evidence from rigorous trials would likely corroborate and reinforce the existing evidence base, a reassurance that could be especially valuable given ongoing attacks on vaccines (29).

## Limitations

Fieldwork, questionnaire administration, and statistical processing for this large-scale CAWI survey were entrusted to a specialized, independent market research agency (Euromedia Research), commissioned at full commercial cost, which applied its own established professional protocols. This arrangement, chosen precisely because the authors' expertise lies in clinical and biomedical science rather than survey methodology, ensured methodological neutrality from the study sponsors, but also means the authors received only the agency's aggregate outputs (cross-tabulated frequencies, percentages, and means) rather than the individual-level dataset. As a result, certain analyses conventionally reported in survey research were not available from the materials provided: cell-level quota fulfillment records, subgroup sample sizes (n) for political-affiliation categories, significance testing, confidence intervals, effect sizes, correction for multiple comparisons, and multivariable regression adjusting for potential confounders. These constraints apply throughout the manuscript and are noted where relevant; smaller political-affiliation subgroups (e.g., minor parties, undecided respondents) should accordingly be read as indicative rather than precise, whereas the larger associations emphasized in the Discussion—consistent in direction and magnitude (often 20–30 percentage points) across nearly all survey items—are unlikely to reflect sampling variability alone.

The completion rate of 33.5% (1,000 completed interviews out of 2,982 contacts) leaves room for potential non-response bias in both directions: parents with stronger pre-existing views on vaccination, whether favorable or critical, were plausibly more likely to complete the questionnaire, which would amplify the polarization observed by political orientation; parents with lower health literacy, conversely, were plausibly under-represented among completers, which would make the knowledge gaps reported here an underestimate rather than an overestimate of those in the general population. Socioeconomic status and educational attainment of non-responders were not collected and cannot be assessed.

As with any cross-sectional survey, causal inferences cannot be drawn from the associations reported, including the association between political affiliation and vaccine-related knowledge and attitudes, which may partly reflect correlated factors such as education, region, or media consumption. Data, including vaccination status, are self-reported; the online (CAWI) methodology may favor respondents comfortable with digital tools; and responses on vaccination, a socially and politically salient topic, may be subject to some degree of social desirability bias. The questionnaire itself was purpose-built for this study, grounded in current Italian legislation and reviewed for comprehensibility prior to administration, though it did not undergo formal psychometric validation. Occupation and self-assessed socioeconomic status were collected (Supplementary material, Q18–Q19) but not analyzed in the present study; educational attainment and household income were not assessed at all. Reporting these characteristics, where available, could add useful context to the interpretation of the findings in future analyses.

These constraints are consistent with the descriptive, hypothesis-generating nature of this survey, which (with a national sample of 1,000 parents) remains, to our knowledge, one of the largest studies to date examining Italian parents' knowledge and attitudes toward childhood vaccination in relation to political orientation.

## Conclusions

In conclusion, the survey highlights an oscillation between two antithetical positions, a mistrust of vaccine (hesitancy) contrasted with a blind trust of vaccine (promptness). The data shows a great deal of misinformation, or knowledge gaps, revealing that the information provided to the population is not commensurate with the importance of the issue; to borrow a metaphor from epidemiology (without implying an established scientific threshold) we could speak of a lack of “herd knowledge”: just as herd immunity requires most of a population to be protected, informed public decision-making arguably requires most parents to hold accurate knowledge, a threshold this survey suggests is far from met.

The public's opinion is based on two key entities, health professionals and the media. In the authors' opinion, both are subject to industry influence, from which they benefit. Until conflicts of interest between these subjects are resolved, achieving the “herd knowledge” needed for a conscious, responsible choice (as required by law under the principle of “informed consent”) will not be achievable. In the authors' opinion, another concern is “regulatory capture,” where the industries or interests dominate regulators, they are supposed to regulate, rather than by the public interest. With this survey, we would like to encourage the provision of accurate information based on serious and thorough scientific work and reliable clinical trials, result of a rediscovered honest scientific dialectic, an essential prerequisite for something that deserves to be defined as “Science.” To address this situation, we propose a structured approach built around a transparent, independent information portal that reports on clinical trials. This portal would serve three main functions: explaining the use of each vaccine in a clear and professional manner, sharing personal experiences from patients and families, and partnering with associations to widen its reach. We also call for active pharmacovigilance. Italy urgently needs this online platform to consolidate these efforts and serve as a hub for independent research, free from the influence of the pharmaceutical industry.

## Data Availability

The aggregate, cross-tabulated tables of the survey results (by parental gender and age, children's age, geographical area, and political affiliation) are available in Zenodo: https://doi.org/10.5281/zenodo.15512208. Individual-level data were not provided to the authors.
