# Supplementary material for: Survey on the knowledge, perceptions, and concerns of parents about childhood vaccinations in Italy
Source: Front Public Health. 2026 Oct 27;14:1950327. doi: 10.3389/fpubh.2026.1950327 (PMC13747952; doi:10.3389/fpubh.2026.1950327)
Supplement: Supplementary file 1 [file Supplementary_file_1.docx]

**Supplementary material**

**Title**: Survey on the knowledge, perceptions and concerns of parents about childhood vaccinations in Italy

**Authors**: Laura Teodori, Eugenio Serravalle, Franco Stocco, Panagis Polykretis

**Supplementary tables**

**Table 1** How important do you think pediatric vaccinations are to the child's health (those to be carried out by the age of 16 years)? — Breakdown by parent's gender, age, children's age, and area of residence.

**Table 1.1** How important do you think pediatric vaccinations are to the child's health (those to be carried out by the age of 16 years)? — Breakdown by political affiliation.

**Table 2** How important do you think pediatric vaccinations are to the community (those to be carried out by the age of 16 years)? — Breakdown by parent's gender, age, children's age, and area of residence.

**Table 2.1** How important do you think pediatric vaccinations are to the community (those to be carried out by the age of 16 years)? — Breakdown by political affiliation.

**Table 3** Are you in favor or against mandatory vaccination against specific diseases for children under the age of 16? — Breakdown by parent's gender, age, children’s age, and area of residence.

**Table 3.1** Are you in favor or against mandatory vaccination against specific diseases for children under the age of 16? — Breakdown by political affiliation.

**Table 4** As far as you know, what are the MANDATORY pediatric vaccinations in Italy? (Multiple answers allowed) — Breakdown by parent's gender, age, children's age, and area of residence.

**Table 4.1** As far as you know, what are the MANDATORY pediatric vaccinations in Italy? (Multiple answers allowed) — Breakdown by political affiliation.

**Table 5** As far as you know, what are the RECOMMENDED pediatric vaccinations in Italy? (Multiple answers allowed) — Breakdown by parent’s gender, age, children's age, and area of residence.

**Table 5.1** As far as you know, what are the RECOMMENDED pediatric vaccinations in Italy? (Multiple answers allowed) — Breakdown by political affiliation.

**Table 6** As far as you know, what are the pediatric vaccinations in Italy...? Summary table (Multiple answers allowed) — Mandatory vs. Recommended.

**Table 6.1** As far as you know, what are the pediatric vaccinations in Italy...? Summary table — Obligation and recommendation rankings (Multiple answers allowed).

**Table 7** Do you think it is necessary to vaccinate your children in order to enroll them in kindergarten, preschool, primary school, first-grade secondary school, and second-grade secondary school? (Multiple answers allowed) — Breakdown by parent’s gender, age, children's age, and area of residence.

**Table 7.1** Do you think it is necessary to vaccinate your children in order to enroll them in kindergarten, preschool, primary school, first-grade secondary school, and second-grade secondary school? (Multiple answers allowed) — Breakdown by political affiliation.

**Table 8** As far as you are aware, what are the mandatory vaccinations to be admitted and attend KINDERGARTEN and PRESCHOOL? (Multiple answers allowed) — Breakdown by parent’s gender, age, children's age, and area of residence.

**Table 8.1** As far as you are aware, what are the mandatory vaccinations to be admitted and attend KINDERGARTEN and PRESCHOOL? (Multiple answers allowed) — Breakdown by political affiliation.

**Table 9** As far as you know, what are the mandatory vaccinations to be admitted and attend PRIMARY SCHOOL (elementary school)? (Multiple answers allowed) — Breakdown by parent's gender, age, children's age, and area of residence.

**Table 9.1** As far as you know, what are the mandatory vaccinations to be admitted and attend PRIMARY SCHOOL (elementary school)? (Multiple answers allowed) — Breakdown by political affiliation.

**Table 10** As far as you are aware, what are the mandatory vaccinations to be admitted and attend FIRST GRADE SECONDARY SCHOOL (middle school)? (Multiple answers allowed) — Breakdown by parent's gender, age, children's age, and area of residence.

**Table 10.1** As far as you are aware, what are the mandatory vaccinations to be admitted and attend FIRST GRADE SECONDARY SCHOOL (middle school)? (Multiple answers allowed) — Breakdown by political affiliation.

**Table 11** As far as you are aware, what are the mandatory vaccinations to be admitted and attend SECOND GRADE SECONDARY SCHOOL (high school)? (Multiple answers allowed) — Breakdown by parent’s gender, age, children's age, and area of residence.

**Table 11.1** As far as you are aware, what are the mandatory vaccinations to be admitted and attend SECOND GRADE SECONDARY SCHOOL (high school)? (Multiple answers allowed) — Breakdown by political affiliation.

**Table 12** As far as you know, what are the mandatory vaccinations to access and attend...? Summary table — Kindergarten/Preschool, Primary School, First and Second Grade Secondary School (Multiple answers allowed).

**Table 13** In your opinion, do the same rules on pediatric vaccinations apply in the rest of Europe as in Italy? — Breakdown by parent’s gender, age, children's age, and area of residence.

**Table 13.1** In your opinion, do the same rules on pediatric vaccinations apply in the rest of Europe as in Italy? — Breakdown by political affiliation.

**Table 14** Do you consider pediatric vaccines a safe health means? — Breakdown by parent’s gender, age, children’s age, and area of residence.

**Table 14.1** Do you consider pediatric vaccines a safe health means? — Breakdown by political affiliation.

**Table 15** How much do you agree with the following statement: “Pediatric vaccines are more dangerous than the diseases to which they are directed”? — Breakdown by parent's gender, age, children's age, and area of residence.

**Table 15.1** How much do you agree with the following statement: “Pediatric vaccines are more dangerous than the diseases to which they are directed”? — Breakdown by political affiliation.

**Questionnaire**

Note: This is the original Italian questionnaire as administered to respondents, including the complete response option lists for every item. For knowledge items (Q5–Q11), the mandatory/recommended status shown in parentheses was not visible to respondents and item order was randomized at each interview.

***Selezione del campione***

*Lei ha almeno un figlio con meno di 17 anni?* [Sì / No – fine intervista]

*Area geografica di residenza dell’intervistato* [Nord Ovest (Valle d'Aosta, Piemonte, Liguria, Lombardia) / Nord Est (Veneto, Trentino Alto Adige, Friuli Venezia Giulia, Emilia Romagna) / Centro (Toscana, Umbria, Marche, Lazio) / Sud (Abruzzo, Molise, Puglia, Basilicata, Campania, Calabria) / Isole (Sardegna, Sicilia)]

**Q1**. *Quanti anni ha/hanno i suoi figli?* (AMMESSE RISPOSTE MULTIPLE) [Da 0 fino a 2 anni compiuti / Da 2 fino a 6 anni compiuti / Da 6 fino a 11 anni compiuti / Dagli 11 fino ai 16 anni compiuti]

**Q2**. *Lei quanto ritiene importanti le vaccinazioni pediatriche (quelle da effettuarsi entro i 16 anni di età) per la salute del bambino?* [Molto / Abbastanza / Poco / Per nulla / Non sa / Non risponde]

**Q3**. *Lei quanto ritiene importanti le vaccinazioni pediatriche (quelle da effettuarsi entro i 16 anni di età) per la comunità?* [Molto / Abbastanza / Poco / Per nulla / Non sa / Non risponde]

**Q4**. *Secondo Lei, nel resto d’Europa valgono le stesse regole in merito alle vaccinazioni pediatriche che valgono in Italia? Esiste, secondo Lei, anche negli altri Stati Europei l’obbligatorietà di alcune vaccinazioni pediatriche come in Italia?* [Sì, nella maggior parte degli Stati europei / Sì, in circa metà degli Stati europei / No, quasi tutti gli Stati europei hanno meno obblighi dell'Italia o nessun obbligo / Non sa / Non risponde]

**Q5**. *Per quello che Lei conosce, quali sono le vaccinazioni pediatriche OBBLIGATORIE in Italia? (AMMESSE RISPOSTE MULTIPLE)*

Nota: Le indicazioni tra parentesi non apparivano sulla schermata degli intervistati; gli item apparivano in ordine randomizzato ad ogni intervista. L'elenco comprendeva 23 voci in totale; le analisi riportate nel manoscritto (Tabelle 4–6) si riferiscono alle 18 vaccinazioni incluse nel Piano Nazionale Prevenzione Vaccinale (obbligatorie o raccomandate in senso stretto), escludendo 5 vaccinazioni raccomandate solo per patologie specifiche o esposizione professionale (anti-herpes zoster, anti-epatite A, anti-meningoencefalite da zecca, anti-tubercolare, anti-poliomielite per condizioni a rischio), riportate nel questionario ma non oggetto di analisi comparativa in questo studio.

[anti-poliomielitica (OBBLIGATORIA) / anti-difterica (OBBLIGATORIA) / anti-tetanica (OBBLIGATORIA) / anti-epatite B (OBBLIGATORIA) / anti-pertosse (OBBLIGATORIA) / anti-Haemophilus influenzae tipo b (OBBLIGATORIA) / anti-morbillo (OBBLIGATORIA) / anti-rosolia (OBBLIGATORIA) / anti-parotite (OBBLIGATORIA) / anti-varicella (OBBLIGATORIA) / anti-meningococcica B (RACCOMANDATA) / anti-meningococcica C (RACCOMANDATA) / anti-pneumococcica (RACCOMANDATA) / anti-rotavirus (RACCOMANDATA) / anti-papillomavirus (anti-HPV) (RACCOMANDATA) / anti-influenzale (RACCOMANDATA) / anti-meningococco quadrivalente ACW135Y (RACCOMANDATA) / anti-herpes zoster (RACCOMANDATA PER PATOLOGIE) / anti-epatite A (RACCOMANDATA PER PATOLOGIE) / anti-meningoencefalite da zecca (RACCOMANDATA PER ESPOSIZIONE PROFESSIONALE) / anti-tubercolare (RACCOMANDATA PER ESPOSIZIONE PROFESSIONALE) / anti-poliomielite (RACCOMANDATA PER CONDIZIONI A RISCHIO) / anti-COVID (RACCOMANDATA PER PATOLOGIE) / Tutte le precedenti / Nessuna delle precedenti / Non sa / Non risponde]

**Q6**. *E, sempre per quello che Lei conosce, quali sono le vaccinazioni pediatriche RACCOMANDATE in Italia?* (AMMESSE RISPOSTE MULTIPLE)

Stessa lista randomizzata della domanda 5; le indicazioni tra parentesi non apparivano sulla schermata degli intervistati.

**Q7**. *Lei crede sia necessario vaccinare i propri figli per poterli iscrivere all’asilo nido, alla scuola materna, alla scuola primaria (scuola elementare), alla scuola secondaria di primo grado (scuola media) e alla scuola secondaria di secondo grado (scuola superiore)?* (AMMESSE FINO A 4 RISPOSTE) [È necessario vaccinarli per iscriverli all’asilo nido e alla scuola materna / È necessario vaccinarli per iscriverli alla scuola primaria (scuola elementare) / È necessario vaccinarli per iscriverli alla secondaria di primo grado (scuola media) / È necessario vaccinarli per iscriverli alla secondaria di secondo grado (scuola superiore) / È necessario vaccinarli per iscriverli ad una qualsiasi scuola di qualunque grado / Non è obbligatorio vaccinare i propri figli per iscriverli in una qualsiasi scuola di qualunque grado / Non sa / Non risponde]

**Q8**. *Per quello che Le è dato di sapere, quali sono le vaccinazioni obbligatorie per accedere e frequentare ASILO-NIDO e SCUOLA MATERNA?* (AMMESSE RISPOSTE MULTIPLE)

Lista analoga alla domanda 5 (23 vaccini + opzioni “*Nessuna*” / “*Tutte le precedenti*” / “*Non sa/Non risponde*”); le indicazioni “NECESSARIA PER ESSERE AMMESSI” tra parentesi non apparivano sulla schermata degli intervistati.

**Q9**. *Per quello che Le è dato di sapere, quali sono le vaccinazioni necessarie per accedere e frequentare LA SCUOLA PRIMARIA (scuola elementare)?* (AMMESSE RISPOSTE MULTIPLE)

Stessa struttura della domanda 8. Agli intervistati veniva inoltre mostrata la seguente nota informativa: “Nessuna vaccinazione è obbligatoria per essere ammessi: i bambini possono accedere comunque a scuola e seguire le lezioni, ma, in caso non siano stati rispettati gli obblighi, viene attivato dalla Asl un percorso di recupero della vaccinazione ed è possibile incorrere in sanzioni amministrative da 100 a 500 euro.”

**Q10**. *Per quello che Le è dato di sapere, quali sono le vaccinazioni necessarie per accedere e frequentare LA SCUOLA SECONDARIA DI PRIMO GRADO (scuola media)?* (AMMESSE RISPOSTE MULTIPLE)

Stessa struttura e stessa nota informativa della domanda 9, riferita alla scuola secondaria di primo grado.

**Q11**. Per quello che Le è dato di sapere, quali sono le vaccinazioni necessarie per accedere e frequentare LA SCUOLA SECONDARIA DI SECONDO GRADO (scuola superiore)? (AMMESSE RISPOSTE MULTIPLE)

Stessa struttura e stessa nota informativa della domanda 9, riferita alla scuola secondaria di secondo grado.

**Q12**. *Lei è favorevole o contrario all'obbligo di vaccinazione contro alcune malattie per i ragazzi minori di 16 anni?* [Sono favorevole e vorrei che diventassero obbligatorie anche altre vaccinazioni oltre a quelle già stabilite per legge (le vaccinazioni raccomandate) / Sono favorevole alle vaccinazioni obbligatorie che attualmente la legge stabilisce / Sono contrario e vorrei che le vaccinazioni fossero solamente raccomandate e non obbligatorie (ogni famiglia dovrebbe decidere autonomamente) / Sono contrario a qualsiasi tipo di vaccinazione / Non sa / Non risponde]

**Q13**. *Lei ritiene i vaccini pediatrici uno strumento sanitario sicuro?* [Sì, in generale sono sicuri / No, molto spesso causano problemi o hanno importanti effetti collaterali / Non sa / Non risponde]

**Q14**. *“I vaccini sono più pericolosi delle malattie verso cui sono indirizzati”. Lei quanto condivide questa affermazione?* [Molto / Abbastanza / Poco / Per nulla / Non sa / Non risponde]

**Q15**. *Per quello che le è dato di sapere, la Costituzione Italiana prevede possibili obblighi di legge per un trattamento sanitario?* [Sì, se non serve solo a migliorare o preservare la salute di chi lo riceve, ma anche quella degli altri / Sì, se serve a migliorare o preservare la salute di chi lo riceve, anche se non serve agli altri / Sì, se serve agli altri, anche se non serve a chi lo riceve / Sì, se serve agli altri, anche se non serve a chi lo riceve, anzi lo può esporre anche a conseguenze non temporanee né di scarsa entità / No, mai / Non sa / Non risponde]

***Anagrafica***

**Q16**. *Genere dell’intervistato* [Maschio / Femmina]

**Q17**. *Età dell'intervistato* [18-24 anni / 25-44 anni / 45-64 anni / Più di 65 anni]

**Q18**. *Professione* [Lavoratori autonomi (Partite IVA, Liberi professionisti, Imprenditori...) / Lavoratori dipendenti (Operai, Impiegati, Docenti, Dirigenti...) / Disoccupati e in cerca di prima occupazione / Studenti / Casalinghe / Pensionati]

**Q19**. *Collocazione socio-economica dell’intervistato* [Alta / Medio-alta / Media / Medio-bassa / Bassa]

**Q20**. *Collocazione politica dell’intervistato* [Fratelli d'Italia-Giorgia Meloni / Lega-Salvini premier / Forza Italia-Berlusconi Presidente / Noi Moderati / Partito Democratico-Italia Democratica e Progressista / Alleanza Verdi e Sinistra / Movimento 5 stelle-2050 #pace / Italia Viva-Renzi / +Europa con Emma Bonino / Azione Calenda Siamo Europei / Sud chiama Nord Cateno De Luca / Pace Terra Dignità / Rassemblement Valdôtain / Südtiroler Volkspartei (SVP) / Alternativa popolare-PPE / Democrazia sovrana e popolare / Partito animalista - Italexit per l'Italia / Altro (specificare) / Scheda nulla / bianca / Non andrei a votare / Indeciso]

This section reports the English translation of the questionnaire. To avoid redundant repetition of lengthy multiple-choice lists (in particular the 23-item vaccine checklist used in Q5–Q11), each translated question below is followed by a bracketed summary of its response format (e.g., single choice, multiple choice, rating scale) rather than a full re-transcription of every response option.

***Sample selection***

*Do you have at least one child under 17 years of age?* [Yes / No – end of interview]

*Geographic area of residence* [single choice: Northwest / Northeast / Center / South / Islands]

**Q1**. *How old is/are your child(ren)?* [multiple choice: age brackets 0–2 / 2–6 / 6–11 / 11–16 years]

**Q2**. *How important do you consider pediatric vaccinations (to be administered by age 16) for your child’s health?* [single choice, 4-point scale: A lot / Quite a bit / A little / Not at all — plus Don’t know/No answer]

**Q3**. *How important do you consider pediatric vaccinations (to be administered by age 16) for the community?* [same 4-point scale as Q2]

**Q4**. I*n your opinion, do the same rules regarding pediatric vaccinations that apply in Italy also apply in the rest of Europe? Do other European countries also have mandatory pediatric vaccination requirements, as in Italy?* [single choice: Yes, in most European countries / Yes, in about half of European countries / No, most European countries have fewer or no obligations compared to Italy / Don’t know/No answer]

**Q5**. *To the best of your knowledge, which pediatric vaccinations are MANDATORY in Italy?* [multiple choice from a randomized list of 23 vaccine items (including 5 items recommended only for specific pathologies or occupational exposure, not included in the manuscript's analysis tables), plus “All of the above” / “None of the above” / Don’t know/No answer; mandatory/recommended status not shown to respondents]

**Q6**. *And, again to the best of your knowledge, which pediatric vaccinations are RECOMMENDED in Italy?* [same randomized list and response format as Q5]

**Q7**. *Do you believe it is necessary to vaccinate your children in order to enroll them in nursery school, preschool, primary school, lower secondary school, and upper secondary school?* [multiple choice, up to 4 selections, across the different school levels, plus “Not mandatory for any school level” / Don’t know/No answer]

**Q8**. To the best of your knowledge, which vaccinations are mandatory to access and attend NURSERY SCHOOL and PRESCHOOL? [multiple choice from the same 23-item vaccine list, plus “None” / “All of the above” / Don’t know/No answer]

**Q9**. *To the best of your knowledge, which vaccinations are necessary to access and attend PRIMARY SCHOOL?* [same format as Q8; an explanatory note on the administrative-recovery procedure and fines for unmet requirements was shown to respondents]

**Q10**. *To the best of your knowledge, which vaccinations are necessary to access and attend LOWER SECONDARY SCHOOL?* [same format as Q9]

**Q11**. *To the best of your knowledge, which vaccinations are necessary to access and attend UPPER SECONDARY SCHOOL?* [same format as Q9]

**Q12**. *Are you in favor of or opposed to mandatory vaccination against certain diseases for minors under 16?* [single choice: In favor, and would extend mandatory status to currently recommended vaccines / In favor of currently mandatory vaccines only / Opposed, prefer recommended-only with family choice / Opposed to any vaccination / Don’t know/No answer]

**Q13**. Do you consider pediatric vaccines a safe healthcare tool? [single choice: Yes, generally safe / No, they often cause problems or have significant side effects / Don’t know/No answer]

**Q14**. *“Vaccines are more dangerous than the diseases they target.” How much do you agree with this statement?* [single choice, 4-point agreement scale: A lot / Quite a bit / A little / Not at all — plus Don't know/No answer]

**Q15**. *To the best of your knowledge, does the Italian Constitution allow for mandatory legal health treatments?* [single choice, 4 conditional-agreement options ranging from “benefits others” to “benefits others even at the respondent’s own risk” / No, never / Don’t know/No answer]

***Demographics***

**Q16**. *Respondent’s gender* [Male / Female]

**Q17**. *Respondent’s age* [bracket: 18–24 / 25–44 / 45–64 / 65+]

**Q18**. *Occupation* [categorical: self-employed / employed / unemployed / student / homemaker / retired]

**Q19**. *Respondent’s socioeconomic status (self-assessed)* [5-point scale: High / Medium-high / Medium / Medium-low / Low]

**Q20**. *Respondent’s political affiliation* [single choice from a list of Italian political parties/coalitions, plus “Other (specify)” / Blank-spoiled ballot / Would not vote / Undecided]

**Supplementary references**

**Tetanus**

Gentili G, R. D'Amelio, M. Wirz, et al. Prevalence of hyperimmunization against tetanus in Italians born after the introduction of mandatory vaccination of children with tetanus toxoid in 1968, Infection 1993;21:80-82.

**Diphtheria**

Bergamini et al. Prevalence of diphtheria toxin antibodies in human sera from a cross-section of the Italian population. Vaccine. 1999;17:286–290.

Bellavite P. Factors that influenced the historical trends of tetanus and diphtheria Vaccine Volume 36, Issue 37, 5 September 2018, Page 5506 <https://doi.org/10.1016/j.vaccine.2018.07.061>.

Anggraeni W, Firdausiah M, Perdana MI. Forecasting the Case Number of Infectious Diseases Using Type-2 Fuzzy Logic for a Diphtheria Case Study. Engineering Proceedings. 2023; 39(1):3.

**Poliomyelitis**

Poliomyelitis prevention: recommendations for use of inactivated poliovirus vaccine and live oral poliovirus vaccine. American Academy of Pediatrics Committee on Infectious Diseases, in Pediatrics, vol. 99, n. 2, 1997, pp. 300–5, DOI:10.1542/peds.99.2.300, PMID 9024465.

Devaux CA et al Is it time to switch to a formulation other than the live attenuated poliovirus vaccine to prevent poliomyelitis?Frontiersn <https://doi.org/10.3389/fpubh.2023.1284337>.

Lahariya C. A brief history of vaccines & vaccination in India. Indian J Med Res 2014;139: 491-511.

Lopalco PL. Wild and vaccine-derived poliovirus circulation, and implications for polio eradication. Epidemiol. Infect., 145 (2017), pp. 413-419.

**Pertussis**

Donzelli A, Bellavite P, Demicheli V Epidemiology of pertussis and prevention strategies: problems and perspectives E&P 2019, 43 (1) gennaio-febbraio p. 83-91 DOI: <https://doi.org/10.19191/EP19.1.A001>.

Sealey KL, Belcher T, Preston A. Bordetella pertussis epidemiology and evolution in the light of pertussis resurgence. Infect Genet Evol 2016;40:136-43.

Klein NP, Bartlett J, Fireman B et al. Waning protection following 5 doses of a 3-component diphtheria, tetanus, and acellular pertussis vaccine. Vaccine 2017; 35(26):3395-400.

Wendelboe AM, Van Rie A, Salmaso S, Englund JA. Duration of immunity against pertussis after natural infection or vaccination. Pediatr Infect Dis J 2005;24%) Suppl: S58-61.

Wearing HJ, Rohani P. Estimating the duration of pertussis immunity using epide­miological signatures. PLoS Pathog 2009;5(10):e1000647.

Althouse BM, Scarpino SV. Asymptomatic transmission and the resurgence of Bordetella pertussis. BMC Med 2015;13:146.

Edwards KM, Berbers GA. Immune responses to pertussis vaccines and disease. J Infect Dis 2014;209:S10-S15.

Warfel JM, Edwards KM. Pertussis vaccines and the challenge of inducing durable immunity. Curr. Opin Immunol 2015;35:48-54.

**Mumps**

Briss PA, Fehrs LJ, Parker RA, et al. Sustained transmission of mumps in a highly vaccinated population: assessment of primary vaccine failure and waning vaccine-induced immunity. J Infect Dis 1994;169:77-82.

Hersh BS, Fine PE, Kent WK, et al. Mumps outbreak in a highly vaccinated population. J Pediatr 1991;119:187-.

Davidkin I, Jokinen S, Broman M, et al. Persistence of Measles, Mumps, and Rubella Antibodies in an MMR-Vaccinated Cohort: A 20-Year Follow-up. JID 2008:197:950-6.

Vygen S, Fischer A, Meurice L, et al. Waning immunity against mumps in vaccinated young adults, France 2013. Euro Surveill 2016;2:30156.

**Haemophilus Influenza B**

Adama HJ, Richardson SE, Jamieson FB, et al. Changing epidemiology of invasive Haemophilus influenzae in Ontario, Canada: Evidence for herd effects and strain replacement due to Hib vaccination. Vaccine 2010;28:4073–78.

André JB, Gandon S. Vaccination, within-host dynamics, and virulence evolution. Evolution 2006;60:13–23.

Dworkin MS, Park L, Borchardt SM. The Changing Epidemiology of Invasive Haemophilus influenzae Disease, Especially in Persons 65 Years Old. Clin Infect Dis 2007;44:810-16.

Gandon S, Mackinnon MJ, Nee S, et al. Imperfect vaccines and the evolution of pathogen virulence. Nature 2001;414:751-6.

Gandon S, Mackinnon MJ, Nee S, et al. Imperfect vaccination: some epidemiological and evolutionary consequences. Proc Biol Sci 2003;270:1129-36.

Gandon S, Day T. Evolutionary epidemiology of vaccination. J R Soc Interface 2007;4:803-17. Soeters H et al Current Epidemiology and Trends in Invasive Haemophilus influenzae Disease—United States, 2009–2015 Clinical Infectious Diseases® 2018;67(6):881–9.

Zanella RC, Bokermann S, Andrade AL, et al. Changes in serotype distribution of Haemophilus influenzae meningitis isolates identified through laboratory-based surveillance following routine childhood vaccination against H. influenzae type b in Brazil. Vaccine 2011;29:8937–42.

**Hepatitis B**

CDC Epidemiology and prevention of vaccine-preventable diseases.14th ed. Hall E, et al. 2021. <https://www.cdc.gov/vaccines/pubs/pinkbook/downloads/hepb.pdf>.

Schillie S, et al. Prevention of hepatitis B virus infection in the United States: recommendation of the Advisory Committee on Immunization Practices. MMWR Recomm Rep. 2018 Jan 12;67(1):3.

Alter MJ, et al. The changing epidemiology of hepatitis B in the United States. Need for alternative vaccination strategies. JAMA. 1990 Mar 2; 263(9):1222.

Mikaeloff Y, Caridade G, Suissa S, et al. Hepatitis B vaccine and the risk of CNS inflammatory demyelination in childhood. Neurology 2009;72:873-80.

**Chickenpox**

Donzelli A, Demicheli V. Vaccinazione antivaricella: argomenti scientifici per possibili strategie diverse dalle attuali. Epidemiol Prev 2018; 42: 65-70.

Edmunson W.J Brisson M, Gay NJ, et al. Varicella vaccination: a double edged sword? Commun Dis Public Health 2002;5:185-6.

Fine PE. Herd immunity: history, theory, practice. Epidemiol Rev 1993;15:265-302 Rodpothong P, Auewarakul P. Viral evolution and transmission effectiveness. World J Virol 2012; 1: 131-4.

**General**

Fine PE. Herd immunity: history, theory, practice. Epidemiol Rev 1993;15:265-302 Rodpothong P, Auewarakul P. Viral evolution and transmission effectiveness. World J Virol 2012; 1: 131-4.
